# Effective connectivity and criminal sentencing decisions: dynamic causal models in laypersons and legal experts

**DOI:** 10.1093/cercor/bhab484

**Published:** 2022-01-18

**Authors:** Takeshi Asamizuya, Hiroharu Saito, Ryosuke Higuchi, Go Naruse, Shozo Ota, Junko Kato

**Affiliations:** Graduate School of Law and Politics, The University of Tokyo, Bunkyo-ku, Tokyo 113-0035, Japan; Center for Evolutionary Cognitive Sciences, Graduate School of Arts and Sciences, The University of Tokyo, Meguro-ku, Tokyo 153-0041, Japan; Graduate School of Law and Politics, The University of Tokyo, Bunkyo-ku, Tokyo 113-0035, Japan; Institute of Social Science, The University of Tokyo, Bunkyo-ku, Tokyo 113-0035, Japan; Graduate School of Law and Politics, The University of Tokyo, Bunkyo-ku, Tokyo 113-0035, Japan; Graduate School of Law and Politics, The University of Tokyo, Bunkyo-ku, Tokyo 113-0035, Japan; Graduate School of Law and Politics, The University of Tokyo, Bunkyo-ku, Tokyo 113-0035, Japan; School of Law, Meiji University, Chiyoda-ku, Tokyo 101-0062, Japan; Graduate School of Law and Politics, The University of Tokyo, Bunkyo-ku, Tokyo 113-0035, Japan; Institute for Diversity and Adaptation of Human Mind, The University of Tokyo, Bunkyo-ku, Tokyo 113-0035, Japan

**Keywords:** dynamic causal modeling, functional connectivity, prefrontal cortex, insula, neurolaw

## Abstract

This magnetic resonance imaging study is designed to obtain relevant implications for criminal justice and explores the effective connectivity underlying expertise. Laypersons and experts considered sentences for remorseful and remorseless defendants, respectively, with and without mitigation, in hypothetical murder cases. Two groups revealed no differential activation. However, dynamic causal modeling analysis found distinct patterns of connectivity associated with subjects’ expertise and mitigating factors. In sentencing for remorseful defendants, laypersons showed increased strength in all bidirectional connections among activated regions of Brodmann area (BA) 32, BA23, the right posterior insula, and the precuneus. In contrast, legal experts sentenced based on mitigation reasoning, showed increased strength only in the bidirectional connection between the insula and the precuneus. When sentencing for remorseless ones without mitigation, both laypersons and experts increased the connection strength, but with reverse directionality, between regions; legal experts strengthened connectivity from BA10 to other regions, that is, the right anterior insula and BA23, but the directionality was reversed in laypersons. In addition, the strength of connection to BA32 and BA10 was correlated with changes in punishments by mitigating factors. This is a crucial result that establishes the validity of the connectivity estimates, which were uninformed by the independent (behavioral) differences in the severity of punishment.

## Introduction

Judgment based on expertise is considered to strengthen rationality and logical consistency in human decisions, and expert judgment often conflicts with lay judgment. One prominent example is found in criminal justice systems; legal experts have the primary role in criminal justice, but some legal systems also include lay judges. Sentencing is based on consideration of the facts and consequences of crimes (Anleu and Kathy 2021). However, law scholars increasingly believe that emotion should be proactively regulated rather than avoided (Abrams and Keren 2010; Maroney 2011; Maroney 2018). This view is among the motives for the introduction of the lay judge system.

The close relationship between reason and emotion in human decisions has also long attracted an attention in neurosciences (Huebner et al. 2009). For example, neural correlates of social and moral cognition are found in regions that are linked to emotion, that is the orbitofrontal cortex, insula, amygdala, temporal parietal junction, and precuneus, as well as cognitive control, typically Brodmann area (BA) 8, BA9, BA10, and BA32 (Forbes and Grafman 2010; Buckholtz and Marois 2012; Van Bavel et al. 2015; Decety and Yoder 2017). Moreover, the recent studies reported connectivity between the prefrontal cortex (PFC) and regions linked to emotion and mentalizing (Li et al. 2014; Jung et al. 2016). Our functional magnetic resonance imaging (fMRI) experiment aimed to bridge two distant disciplines, law and neuroscience, with an interest in the relationship between reason and emotion. For this purpose, we examined the effective connectivity involved in the application of legal expertise.

**Figure 1:**
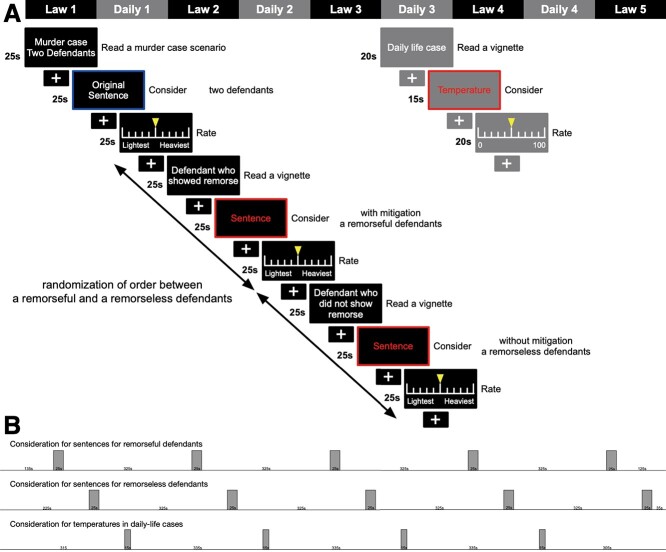
(*A*) Task design; (*B*) regressors of GLM. In each of five law tasks, subject repeatedly read a murder case scenario and two vignettes about remorseful and remorseless defendants; consider what they had read; and sentence the defendants. First, subjects were required to read a murder case in which two defendants, A and B, were equally involved in a murder, and were thus jointly accused of committing it; subjects were then required to decide on each defendant’s punishment. Then, they read a follow-up vignette on one remorseful and one remorseless defendant in a random order and updated their decision on each defendant’s punishment. In one vignette, the remorseful defendant confessed her guilt in the commission of the murder. She gave a sincere apology and even offered compensation to the victim’s family. In another vignette, the remorseless one argued that the remorseful one had acted alone in the commission of the crime. She added that the victim had resisted and was thus killed accidentally. The analysis focused on brain activation as subjects considered the severity of the punishment of each of the defendants (indicated in red). The contrast was obtained by subtracting daily-life brain activity (elicited when subjects considered temperatures) from the activity that occurred in the legal context of considering a sentence (indicated inred).

The experimental framework is based on a critical problem in criminal justice, that is, the consideration of defendants’ remorse in sentencing. Remorse is not specified in the text of the penal code (Bibas and Bierschbach 2004) (see also Supplementary Material) but is expected to heal the psychic wounds of victims and bereaved families, reconcile the damage to society and increase the likelihood of the defendant’s reform and rehabilitation. Here, causal-intentional reasoning is closely related to consideration for morally relevant actions in which emotion is implicated. Judges often weigh remorse and apologies from defendants at sentencing and mitigate their punishment accordingly. Building on real practice in criminal justice, our experiment recruited both laypersons and legal experts under experimental control. Experimental control also defined two different legal judgments, that is, sentencing decisions for defendants who had the same degree of involvement in a murder, with one showing remorse and the other remaining remorseless (Fig. 1*A*). These decisions appear symmetric but are different in nature when they are considered in reference to legal reasoning. Decreasing the sentence for remorseful defendants is based on legal reasoning about mitigating factors, but increasing the sentence for remorseless defendants lacks a reasoning on which legal judgment is based. Therefore, a sentence without mitigating factors is considered more sensitive and challenging in legal practice than one with mitigating factors. The analyses thus interrogated the neural processes of both laypersons and legal experts in two conditions when they considered appropriate punishments for remorseful and remorseless defendants, that is, sentencing with and without mitigation, respectively (2 × 2 experimental design in Table 1). Based on the design, we examined following hypotheses:

**Table 1:** Experimental design

Remorse of defendants	Expertise of subjects	
	Laypersons	Legal experts
Remorseful	Laypersons with mitigation	Legal experts with mitigation
Remorseless	Laypersons without mitigation	Legal experts without mitigation

H1: Decisions to sentence with and without mitigation involve distinct brain process and activated different regions.

H2: Laypersons and legal experts observe distinct patterns of effective connectivity across regions linked to each sentencing decision with and without mitigation.

The neural correlates of sentencing include both regions linked to cognitive control and regions linked to emotion, as expected from the existing studies on human social and moral judgment. Whole-brain general linear model (GLM) analyses (Fig. 1*B*) revealed no differential activation between laypersons and experts but found significant activation linked to different regions between two sentencing decisions. BA32, precuneus, and the right insula were activated when both groups of subjects considered punishments for a remorseful defendant, that is, sentencing with mitigation. In addition to these four regions, two regions, the left anterior insula and BA10, were also activated when increasing punishment for a remorseless defendant, that is, more difficult consideration to sentence without mitigation.

We then applied dynamic causal modeling (DCM) analysis (Stephan et al. 2010; Friston et al. 2019) to the activated regions in each sentencing decision for causal interpretation and found a significant difference between laypersons and legal experts in effective connectivity. DCM analysis provides estimates of effective connectivity; namely, the directed and usually reciprocal connectivity between two regions that underwrites the causal influence of one region on the other. This should be contrasted with functional connectivity that simply reports the (undirected) correlations between two time series. Assessing functional brain architectures with effective connectivity in this way has been used in many settings, including the connectivity architectures associated with social decisions (Van Overwalle et al. 2020).

When making sentencing decisions with mitigating factors (for a remorseful defendant), laypersons showed strengthening of all bidirectional connections across activated regions, but legal experts showed strengthening of only one bilateral connection between the precuneus and the right insula. In contrast, the directionality but not the intensity of connections differed in sentencing decisions without mitigating factors (for a remorseless defendant). Legal experts exhibited strengthened connectivity from the PFC (BA10) to the right anterior insula, and laypersons showed strengthening of the reverse connection. Overall, the analysis revealed the effective connectivity that underlying subjects’ legal expertise. Legal experts showed strengthened connectivity from BA10 to emotion-related regions, that is, the right anterior insula and BA23, only for the more difficult decision, that is, sentencing without mitigating factors. In contrast, among laypersons, regardless of the presence or absence of mitigating factors, the right insula showed strengthened connectivity with other regions including the PFC, that is, BA32 and BA10, respectively. Here, sentencing caused experts to show strengthened connectivity from regions linked to cognitive control, specifically, BA10, whereas laypersons showed strengthened connectivity from the emotion-related regions, specifically, the right insula.

Connectivity analysis between the PFC and the insula reveals the way in which legal expertise is implicated in criminal sentencing. These findings clarify a difference between laypersons and legal experts in a context that is relevant to real practice in criminal justice. The implications of these neural mechanisms can help to clarify the differences in legal decision-making between laypersons and legal experts and inform policy decisions on the participation of lay judges in criminal sentencing.

## Materials and Methods

### Participants

Sixty-six right-handed healthy subjects, including 28 laypersons and 38 legal experts, participated in this study. All subjects had normal or corrected-to-normal vision and no history of neurological or psychiatric damages, illnesses, and disorders. Twenty-eight laypersons were recruited on the university campus after confirmation that they were not specializing in law (nine females; mean age = 21.32 ± 2.36 [standard deviation, SD] years; ranging from 18 to 29 years). The ages of the experts were higher than those of laypersons (i.e., nonlaw students), because several law practitioners were also recruited with law students who had just passed the bar exam. Among 38 legal experts, we recruited 30 university students from the pool of recent successful applicants of the National Bar Examination (8 females; mean age = 25.00 ± 1.23 [SD] years; ranging from 23 to 27 years), and 8 experienced law practitioners (3 females; mean age = 40.88 ± 9.25 [SD] years; ranging from 34 to 63 years). With this composition of the expert group, we examined whether the results of the expert group were different because of the practical experience in law. Since the ages of the laypersons were not very much different from those of the law students, we could exclude the possible effect of age differences if finding the same results between law students and law practitioners. Four additional individuals participated in the study, but their data were excluded because of problems during the data normalization process. The Ethics Committee of the university approved the study. Written informed consent was obtained from all subjects prior to participation in the study, and all subjects were compensated for their participation.

### Stimuli and Task

The experiment ensured that both subjects, that is, laypersons and legal experts, would parse sentences for remorseful versus remorseless defendants, along with an experimental control that changed only the context or cognitive set (Table 1). For this purpose, the task was designed as follows. Inside an MRI scanner, subjects were asked to read a case in which two defendants had the same degree of involvement in a murder and to decide the severity of the defendants’ punishment. After sentencing them (shown in blue in Law session in Fig. 1*A*), subjects were shown follow-up vignettes in which a given defendant did or did not express sincere remorse, and the subjects were asked to consider the sentencing again and update their decision for each defendant (sentences with and without mitigation, respectively, for remorseful and remorseless defendants, shown in red in Law session in Fig. 1*A*). The order of the sentencing tasks with and without mitigating factors was randomized. In the same murder case, each subject decided the severity of punishments three times: original sentence for two defendants, and then sentences for the remorseful and remorseless defendants, respectively.

Criminal law specialists (among the co-authors) created five hypothetical cases of robbery murders. Each scenario was prepared such that the seriousness of the crimes and the intention (“mens rea”) and legal responsibility of the defendants were the same, although they differed in the facts (when, where, and who) and circumstances (the reason why defendants killed the victim) of the murder. This ensured that subjects understood that two defendants (conspirators) were involved to equal degrees in the murder of a victim based on criminal cases with sufficient evidence (i.e., with no reasonable doubt of the facts) in each. The same specialists also created vignettes in which one defendant, but not the other defendant, showed remorse and apologized after they committed the crime. In judging the severity of punishments, subjects were directed to rate the appropriate criminal sentences for the defendants using a scale from 0 (labeled “lightest criminal punishment”) to 100 (labeled “heaviest criminal punishment”). This scale was chosen over other possible response scales, such as the length of a prison sentence, so that the answers would not be affected by the subjects’ difference in legal knowledge, since the study was focused on comparison between laypersons and legal experts.

Between each of the five legal decision-making sessions (murder cases), presented in random order, we inserted four sessions of daily-life tasks, in which subjects were asked to read commonplace scenarios and rate the Celsius temperature of the item or process in it (e.g., brewing coffee). These sessions were included to contrast with the brain activation associated with the legal decision-making sessions, as explained below. Details are explained in Supplementary Text and Supplementary Figure A.

### Sentencing Data on the Severity of Punishment

As explained above, subjects decided the severity of punishment three times in a murder case; thus, there were three types of sentencing data: the original sentence for two defendants; the sentence for the remorseful defendant; and the sentence for the remorseless defendant.

Since the analysis was focused on changes in punishment in response to mitigating factors, we calculated the following values from the sentencing data and examined their correlations with the neural data:

Punishment reduction = [original sentence] − [sentence for a remorseful defendant].

Punishment increase = [sentence for a remorseless defendant] − [original sentence].

Difference in punishment = [sentence for a remorseless defendant] − [sentence for a remorseful defendant].

### MRI Data Acquisition

The 3D *T*_1_-weighted structural images and multislice *T*_2_-weighted echo-planar volumes with blood oxygen level dependent (BOLD) contrast (51 axial slices with a voxel resolution of 3 × 3 × 3 mm covering the whole brain (repetition time [TR] = 1 s; echo time [TE] = 27 ms; acquisition time [TA] = 0.94 s; acceleration factor = 3, multiband accelerated echo planar imaging (EPI) pulse sequence by the Center for Magnetic Resonance Research [Department of Radiology, University of Minnesota]) were obtained using a 3.0 T MRI scanner (Siemens Prisma). Functional imaging data were acquired in a single scanning session that lasted approximately 34 min 10 s each, in which 2050 volumes were obtained. A *T*_1_-weighted anatomical image lasting 4 min 40 s was acquired before the functional sessions for each participant.

### Physiological Data Acquisition

Pulse and respiration were measured simultaneously with the EPI scan for RETROICOR correction.

### fMRI Data Processing

We performed RETROICOR correction (Glover et al. 2000) on multiband EPI data. RETROICOR-corrected fMRI data were analyzed using Statistical Parametric Mapping (SPM12b for the GLM and DCM, www.fil.ion.ucl.ac.uk/spm12). In summary, EPI images were realigned, slice-timing corrected, co-registered with *T*_1_-weighted structure images and normalized to a standard EPI template based on the Montreal Neurological Institute (MNI) reference brain. The resulting images were spatially smoothed using a 6-mm Gaussian kernel.

### General Linear Model

The time series in the first-level analysis were modeled using boxcar regressor task blocks. Appropriate stimulus functions were convolved with the canonical hemodynamic response function to form regressors. A GLM was used to estimate the parameters for each task: law (murder cases) and daily-life scenarios. To detect brain activity specific to sentencing in law, activation was compared with activation when participants rated the Celsius temperature of the object or process in daily life. In summary, we first identified brain regions engaged by legal decision-making by evaluating the significance of a contrast comparing mitigated and unmitigated legal decision-making (i.e., sentencing for remorseful and remorseless defendants) against nonlegal decision-making (i.e., making decisions about temperature in daily-life sessions). This contrast compared the activations due to distinct cognitive sets modeled as boxcar regressors. These regressors correspond to the red blocks in Figure 1*A* and are listed in Figure 1*B*. For example, the two legal decision-making blocks comprised boxcar functions lasting for 25 s at the appropriate points in the scanning session. In these statistical parametric mapping analyses, we modeled the entire time series for each subject and then used a standard summary statistic procedure to identify significant differences in brain responses over subjects—by testing for differences in the appropriate parameter estimates at the between subject level. We principally applied a threshold of family-wide error (FWE)-corrected *P* < 0.05 at a voxel or cluster level for multiple comparisons across the whole brain.

### Dynamic Causal Modeling

This method allows estimations of causal inference between neuronal responses and infer distinct connectivity (Friston et al. 2019; Zeidman 2019; Zeidman, Jafarian, Corbin, et al. 2019a; Zeidman, Jafarian, Seghier, et al. 2019b). All DCMs were created, and the data were estimated as deterministic (Daunizeau et al. 2012), bilinear (Stephan et al. 2008), and two-state DCM (Marreiros et al. 2008), with mean-centered inputs to infer the effective connectivity as the best explanation for each subject’s neuroimaging time seriesdata.

### Volume of Interest Extraction

For DCM analysis, volumes of interest (VOIs) were extracted based on the peaks of the above contrasts in the sentencing of remorseful and remorseless defendants using GLM analysis.

Time series from VOIs associated with different contrasts were summarized using the SPM12 Eigenvariate toolbox. We extracted each subject’s principal eigenvariate around the participant-specific local maxima of activation nearest to the peak voxel of the group (second level) GLM analysis (see Table 2). For VOI extraction, the normalized images were smoothed with a 6-mm Gaussian kernel to improve anatomical accuracy. The radius of the VOI spheres was 8 mm, and the search radius for local maxima from the group analysis was restricted to 16 mm. We combined the 8-mm spheres of two peak coordinates for the search in the right anterior insula for the “remorseless defendant” contrast. All voxels contributing to the eigenvariates were significant at *P* < 0.05 uncorrected and adjusted at *P* < 0.05 for the effects of interest (i.e., only for those regressors that were used in the DCMs for input or modulation).

**Table 2:** Activation of the contrast of legal (sentence) > nonlegal (daily life) decisions

(*A*) When considering a sentence for a remorseful defendant (punishment reduction)									
Region	Side	BA	MNI coordinates				Peak level	Cluster level	
			*x*	*y*	*z*	*T*	P_FWE_corr	P_FWE_corr	*k*
Precuneus	R	R BA31	8	−64	32	7.633	0.000	0.000	1812
BA23	R	R BA23	4	−16	32	5.928	0.003	0.000	1812
BA32	L	L BA32	0	38	22	5.087	0.046	0.000	954
Insula [posterior]	R	R BA13	38	−14	2	4.743	0.128	0.000	867
(*B*) When considering a sentence for a remorseless defendant (punishment increase)									
Region	RL	BA	MNI coordinates				Peak level	Cluster level	
			*x*	*y*	*z*	*T*	P_FWE_corr	P_FWE_corr	k=
Precuneus	R	R BA31	8	−62	32	8.404	0.000	0.000	2529
BA23	R	R BA23	2	−18	32	6.054	0.002	0.000	2529
BA32	L	L BA32	2	46	16	7.425	0.000	0.000	2205
BA10	R	R BA10	8	50	2	7.332	0.000	0.000	2205
Insula (dorsal anterior)	R	R BA44	36	8	10	5.234	0.036	0.238	95
Insula (ventral anterior)	L	L BA13	−32	16	−12	4.996	0.076	0.223	98

### Specification of DCMs

Our analysis of effective connectivity used the following procedure. First, we estimated the effective connectivity (changes in connectivity due to legal decision-making encoded by the B matrix in DCM) for each subject using time series that were selected and concatenated to distinguish two tasks decision-making blocs of sentencing with or without mitigation. This meant that we fitted two DCMs to each subject under the two legal decision-making conditions. We used different brain regions or nodes based upon the GLM that identified the responses specific to legal decision-making (see General Linear Model section and Table 1). To establish the predictive validity of the ensuing subject and condition-specific connectivity estimates, we then looked for correlations with the differences in punishments (between remorseful and remorseless defendants) over subjects. Crucially, this provides an independent validation, because the differences in punishment over subjects were independent data that were not used when fitting each subject’sDCM.

### Parametric Empirical Bayes Analysis

At the group (second) level, subject-specific connectivity parameters were evaluated using parametric empirical Bayes (PEB) analysis (Friston et al. 2015; Friston et al. 2016; Zeidman 2019; Zeidman, Jafarian, Seghier, et al. 2019b).

To address differences in functional architectures between mitigated and unmitigated decision-making—and between the two groups—we then pooled the connectivity estimates using PEB. This is just a hierarchical extension of Bayesian modeling that allowed us to estimate condition and group means—and the implicit main effects and interaction in our 2 × 2 design. This procedure allows us to use Bayesian model comparison to assess the main effects of condition (mitigated versus unmitigated), group (laypeople versus experts), and their interaction. The PEB analysis also provides subject and condition-specific estimates, suitably adjusted for the group effects. This enabled us to repeat the validation procedure above by looking for correlations over subjects between connectivity and the changes in punishment using the behavioral measures. Note that because our primary interest was in testing hypotheses about differences in effective connectivity between conditions and groups, we used full connectivity for all four DCM analyses.

## Results

### Behavioral Analyses

**Figure 2:**
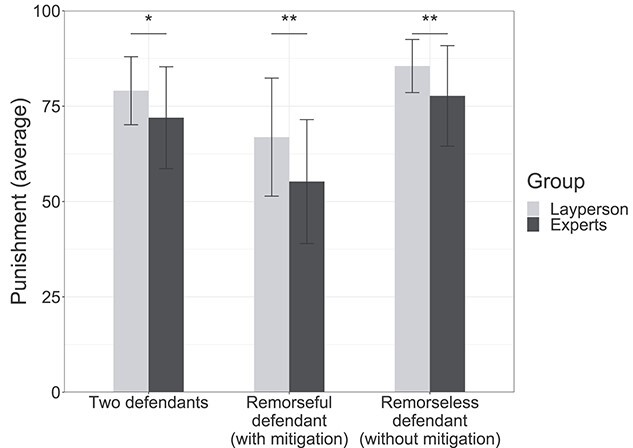
Behavioral analyses. All subjects decreased or increased the severity of punishment for a remorseful or remorseless defendant who did or did not show remorse. Each bar shows the average sentences of each group in each condition. All subjects decreased and increased the punishment of defendants who did and did not, respectively, show remorse. Difference in punishment = [sentence for a remorseless defendant] − [sentence for a remorseful defendant]. Punishment reduction = [original sentence] − [sentence for a remorseful defendant]. Punishment increase = [sentence for a remorseless defendant] − [original sentence].

Laypersons and legal experts were asked to rate the severity of sentencing punishment from 0 (labeled “lightest”) to 100 (labeled “heaviest”) for both defendants and for a remorseful and a remorseless defendant in five fictious/hypothetical murder cases. The averages for each rating in five cases are summarized in Figure 2. No significant differences were found in average ratings across five cases. All subjects, that is, laypersons and experts, always reduced punishment from the original sentence for a remorseful defendant and increased punishment from the original sentence for a remorseless defendant, with no exceptions. Sentences for remorseful and remorseless defendants were significantly different (difference in punishment, *P* = 8.79e−14, *F*_[1, 128]_ = 69.987).

Laypersons imposed significantly heavier punishments than legal experts for all three sentences: the original sentence for two defendants (*P* = 0.0274, *F*_[1, 64]_ = 5.094); “punishment increases” for remorseful defendants (*P* = 0.00958, *F*_[1, 64]_ = 7.134) and “punishment reductions” for remorseless defendants (*P* = 0.00844, *F*_[1, 64]_ = 7.389). The “difference in punishment” was also larger among laypersons than legal experts (*P* = 0.000267, *F*_[1, 128]_ = 14.058). However, as reported above, both groups increased and decreased punishments in the same way, and thus, no interaction was observed between sentencing decisions and groups of laypersons and legal experts.

### Activation in Sentencing with and without Mitigation (Legal > Nonlegal)

**Figure 3:**
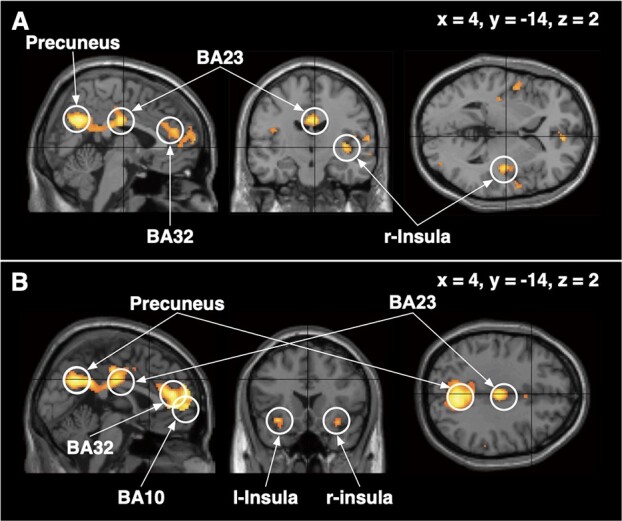
Activation (sentence > temperature) among laypersons and legal experts: (*A*) when sentencing with mitigation for a remorseful defendant and (*B*) when sentencing without mitigation for a remorseless defendant. Activation was obtained after subtracting the contrast of consideration to rate the Celsius temperature in the daily life from the one to rate sentence of a defendant (the contrast of sentence [law] > temperature [daily life]).

GLM analysis using fMRI was performed on brain activity associated with a contrast comparing mitigated and unmitigated legal decision-making (i.e., sentencing for remorseful and remorseless defendants) against nonlegal decision-making (i.e., making decisions about temperature). This contrast compared the activations due to distinct cognitive sets (Table 1) modeled as boxcar regressors. These regressors correspond to the blocks in Figure 1*A* and are listed in Figure 1*B*. This process enabled us to detect activation that was specific to considering criminal sentences. Significant activation using a whole-brain voxel-level FWE-corrected threshold of *P* < 0.05 was found in BA32, BA23, the precuneus, and the right posterior insula when subjects sentenced remorseful defendants with consideration for the mitigating factors (Fig. 3*A* and Table 2*A*). When subjects sentenced remorseless defendants without consideration for mitigating factors, significant activation was found in BA10, BA32, BA23, the precuneus, and the bilateral insula (Fig. 3*B* and Table 2*B*). Between-subjects analysis of variance (ANOVA) showed no significant differential activation between laypersons and legal experts. The Supplementary Table summarizes all activated regions.

### Insula Activation Only among Laypersons Correlated with Sentencing Decisions

**Figure 4:**
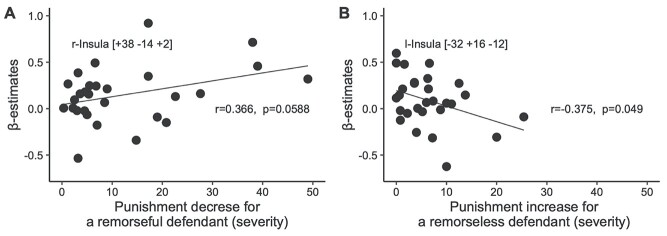
Correlated activation in the insula among laypersons. In laypersons, higher activity in the right posterior insula was associated with a larger difference in punishment between remorseful and remorseless defendants (*A*), but the left anterior insula was activated if laypersons were reluctant to increase punishment for remorseless defendants (*B*).

For laypersons, activation in the right posterior insula (*x* = 38, *y* = −14, *z* = 2) was positively correlated with punishment reduction for remorseful defendants (corr = 0.366, *P* = 0.0588; Fig. 4*A*). However, activation in the left anterior insula (*x* = −32, *y* = 16, *z* = −12) was negatively correlated (corr = −0.375, *P* = 0.049) with an increase in the punishment of a remorseless defendant (Fig. 4*B*). No such correlations were found among legal experts.

### Connectivity Analyses of Laypersons and Legal Experts with and without Mitigation

The connectivity analysis used DCM based on the PEB method (Stephan et al. 2010; Friston et al. 2013). DCM was performed to create two models using VOIs whose peak coordinates were obtained from the activated regions in the GLM analysis for the mitigated and unmitigated legal decisions (>nonlegal, daily-life decisions), as shown in Table 2. We were unable to create VOIs for two and three laypersons from the contrast of reducing a remorseful defendant’s punishment and increasing a remorseless defendant’s punishment, respectively. Four legal experts were excluded for the same reason in two conditions (two of them were excluded from both conditions). Therefore, DCM analysis included 26 (8 females; mean age = 21.42 ± 2.40 [SD] years) and 25 (8 females; mean age = 21.24 ± 2.37 [SD] years) of the 28 laypersons for the conditions of reducing a remorseful defendant’s punishment and increasing a remorseless defendant’s punishment, respectively; 34 of the 38 legal experts in both conditions (Supplementary Material). A full DCM was specified and estimated for each of these subjects.

The first DCM, reflecting sentencing with mitigation for a remorseful defendant included four VOIs: BA32, BA23, the precuneus, and the right posterior insula. The second model, reflecting sentencing without mitigation for a remorseless defendant, included six VOIs: BA10, BA32, BA23, the precuneus, and the right and left anterior insular cortex. Then, we examined whether the effective connectivity, that is, changes in connectivity strength between activated regions, was different depending on the level of subjects’ expertise in law. The two DCMs were thus created by dividing subjects into two groups, that is, laypersons and legal experts in each of legal decisions, that is, decisions with or without mitigation. As a result, four DCMs were conducted along with four cells in Table 1.

We also conducted a separate PEB analysis for each of two decisions with and without mitigation for all subjects to see whether the level of subjects’ expertise in law was examined by a single model (see Supplementary Fig. B for the results of two DCMs for all subjects). The result indicated, but failed to clarify, differences between the two groups. Therefore, we reported the DCM analysis for each of four cells of the 2 × 2 design as a major result.

**Figure 5:**
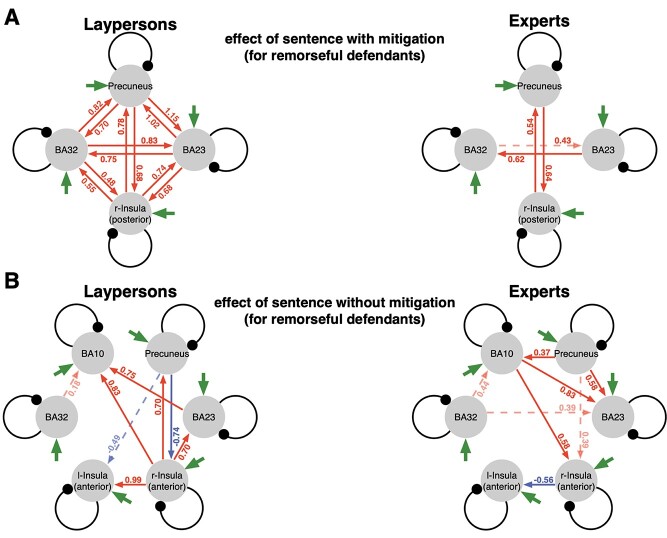
Changes in connectivity due to sentencing (*A*) with mitigation (for a remorseful defendant) and (*B*) without mitigation (for a remorseless defendant). A solid line indicates effective connectivity with a posterior probability >0.95 but is nearly equal to 1.00, and a dotted line indicates effective connectivity with a posterior probability between 0.95 and 0.90. The arrow indicates the direction of effective connectivity from one region to another. Strengthened connections are in red and weakened connections are inblue.

Changes in connectivity of four DCMs are listed in Figure 5. A solid line indicates effective connectivity with a posterior probability >0.95, but it was nearly equal to 1.00, and a dotted line indicates effective connectivity with a posterior probability between 0.95 and 0.90. The arrow indicates the direction of effective connectivity from one region to another. Strengthening of connectivity is shown in red, and weakening of connectivity is shown inblue.

In DCMs of mitigation decision, two groups, that is, laypersons and legal experts, had distinct patterns of changes in connectivity, especially in intensity of connections across four regions. When laypersons sentenced a remorseful defendant with mitigating factors, all connections between all regions exhibited significantly strengthened connectivity in both directions (Fig. 5*A*, laypersons). In contrast, connectivity among legal experts was significantly strengthened only between the precuneus and the right posterior insula and from BA23 to BA32 but not in other regions (Fig. 5*A*, experts). In particular, the connectivity between the right insula and BA32 was strengthened among laypersons but absent among legal experts.

In DCMs of decision without mitigation, not the intensity but direction of connections across six regions was distinct. When sentencing a remorseless defendant without mitigating factors, the connectivity was strengthened in the reverse direction laypersons and legal experts exhibited. Connectivity in laypersons was strengthened with the anterior insula (from the right to left); from the right insula to other regions, including BA10; and from BA23 to BA10. (Fig. 5*B*, laypersons). Connectivity in legal experts was strengthened in the opposite direction of laypersons, that is, from BA10 to the right insula and BA23. Connectivity was also weakened from the right to left insula, which contrasts the strengthened connectivity among laypersons (Fig. 5*B*, experts). Thus, the connectivity from the right to left anterior insula was specific to laypersons. In contrast, the directionality of connectivity from BA10 to the right anterior insula was associated with a higher level of law expertise. Because we used a two-state DCM, all the extrinsic (between regions) connections are positive. This means that changes in connectivity between conditions or groups have the unique interpretation terms of strengthening or weakening, because they can never be less thanzero.

### ANOVA of the Subject-Specific Parameter Estimates from the PEB Analysis

We compared the results among legal experts, that is, between successful applicants of the bar exam (law students) and law practitioners. In mitigation decision for remorseful defendants, we found a significant difference only in subject-specific parameter estimates of connections from the right insula to other regions among which the only one to the precuneus had a posterior probability >0.95 at the group level. No significant differences were found in sentencing for remorseless defendant. This meant that neither the practical experiences nor the ages affected the results. This also refuted the possibility that differences in age affected the results between laypersons and legal experts. As explained in the section on participants, the average age of legal experts was much higher the one of laypersons since the latter included law practitioners (see Supplementary Material).

### Connectivity from an Emotion-Related Region to the Prefrontal Cortex (BA32 and BA10) Correlated with Changes in Punishment

**Figure 6:**
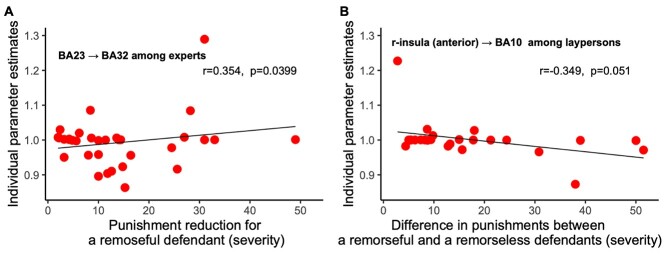
Correlations between connectivity strength and punishment.

We examined whether effective connectivity was related to sentencing decisions by calculating correlations between parameter estimates of connectivity and sentencing decisions. Connectivity from BA23 to BA32 correlated with punishment reduction for a remorseful defendant among experts (corr = 0.354, *P* = 0.0399; Fig. 6*A*), that is, connectivity was strengthened when experts reduced punishment for a remorseful defendant. Connectivity from the right anterior insula to BA10 negatively correlated with the differences in punishment among laypersons (corr = −0.349, *P* = 0.051; Fig. 6*B*), that is, connectivity was weakened when laypersons imposed a larger difference in punishments for remorseful and remorseless defendants. Overall, the effective connectivity from the emotion-related region to the PFC was implicated in decisions to change sentences. A larger change in punishment was associated with strengthened connectivity to BA32 among experts and weakened connectivity to BA10 among laypersons. This correlation with changes by mitigating factors (rather than the level of punishments) is also a crucial result that establishes the validity of the connectivity estimates.

## Discussion

Criminal justice systems in many countries make lay judges participate in sentencing to ensure consideration from different perspective from the one by experts. Specialized knowledge and accumulated experience serve to make experts’ decisions rational. This study examined the brain process linked to legal judgment and then explored the connectivity patterns underlying legal expertise in contrast with the patterns implicated in lay judgment. To ensure the relevance of this study to real practice, the experimental manipulation was focused on the remorse of the defendants, which is taken into consideration for sentencing but also is expected to induce emotional reactions. The GLM analysis found no differential activation between laypersons and legal experts in each sentencing decision. However, the DCM analysis found distinct patterns of effective connectivity in activated regions between laypersons and experts, as well as between condition with and without mitigation. DCM analysis has been used in only a small number of studies on social stimuli (Wang et al. 2017; Ray et al. 2020) but is effective when one cannot make a presumption (before analysis) on connections across activated areas modulated by stimuli and tasks (Hillebrandt et al. 2013; Fastenrath et al. 2014; Tettamanti et al. 2017; Arioli et al. 2018). In this study, the neural mechanism specific to legal expertise was found in effective connectivity but not in the activation of regions, supporting the utility of examining effective connectivity.

Laypersons and legal experts unanimously reduced and increased punishment when they were informed that defendants did and did not show remorse, respectively, and both showed the activation of certain regions, that is, the insula, precuneus, BA32, and BA10 (Table 2; Fig. 3). Neuroscientists have hypothesized that causal-intentional inference in moral and social cognition is closely related to the working of emotion. Law scholars increasingly regard emotion as playing a significant role in deciding punishments that inevitably involve moral considerations, such as considerations taking defendants’ remorse into account. During consideration of sentences with and without mitigation (for remorseful and remorseless defendants), both the ventromedial PFC (vmPFC) and the emotion-related regions were activated. Their co-activation is consistent with widely accepted views in neuroscience andlaw.

The GLM analysis found four regions, that is, the precuneus, BA23, BA32, and the right insula that were activated commonly in sentencing decisions with and without mitigation, but also revealed that the BA10 and the left anterior insula were linked only to decisions without mitigation. This difference in activated regions implies the association of distinct neural process with each of sentencing decisions. In the law discipline, criminal sentences with and without mitigation are considered different decisions in nature, and this may explain the different activation results. Reducing sentences for remorseful defendants is based on reasoning about the mitigating factors, with which legal experts were familiar. Regions of overlapping activation were the precuneus, BA23, BA32, and the right insula. BA23 and the precuneus are anatomically part of the circuits in the anterior and posterior cingulate, which are linked to emotion and memory (Weininger et al. 2019). The insula and precuneus are linked with processing emotion in moral cognition (Garrigan et al. 2016; Zahn et al. 2020). In contrast to these regions, BA10 and the bilateral anterior insula were implicated only in sentencing without mitigation (for remorseless defendants). Increasing sentence for a remorseless defendant is not based directly in legal reasoning and thus requires more careful consideration even among legal experts. The subtlety of the decision might be a reason for the implication of BA10 and the anterior insula.

In the PFC, general reasoning is linked to BA10 and is dissociated from social mentalizing, which is linked to BA32 (Van Overwalle 2011). BA10 is implicated in high-level cognitive integration and in choosing between alternative behaviors, and its functions are specifically important for human cognitive control and emotional regulation (Ramnani and Owen 2004)^,^ (Gilbert et al. 2006). The anterior PFC (encompassing the frontopolar cortex and BA9/10) was more often associated with moral judgments based on facts, in contrast to the anterior cingulate cortex (typically BA32), which was more often associated with moral judgments based on social conditions (Moll et al. 2005). The anterior insula is also concerned with the higher control needed to integrate autonomic and visceral inputs with emotion and motivation because it is mostly connected to limbic regions, such as the vmPFC, that is, BA32 and BA10 (Bernhardt and Singer 2012; Lindquist and Barrett 2012; Nieuwenhuys 2012; Cauda et al. 2013; Sescousse et al. 2013). Overall, sentencing without mitigation (for remorseless defendants) might have activated regions linked to higher cognitive control and general reasoning, in contrast to punishment reductions based on existing reasoning about mitigating factors (reducing the punishment of remorseful defendants).

Although there was no differential activation between two groups in each sentencing decisions, correlated activation was found in the insula with sentencing decisions among laypersons but not among legal experts. More activation was found in the right posterior insula when laypersons imposed more divergent punishments on remorseful and remorseless defendants (Fig. 4*A*). A previous study (Yamada et al. 2012) reported that the right posterior insula was activated by increasing sympathy for defendants with mitigating factors. In contrast, the activation of the left anterior insula was greater when laypersons were less inclined to increase punishment for a remorseless defendant (Fig. 4*B*). The left anterior insula was activated when subjects were instructed to detach themselves from emotional stimuli, for example, to avoid imagining and empathizing the pain of others (Duerden et al. 2013). Sentencing is based on rational considerations of fact, but it is also affected by emotion when moral considerations are involved. The result might have suggested that emotion was implicated more closely with sentencing decisions among laypersons than among legal experts.

Moreover, the analysis revealed a distinct pattern of connectivity between two groups in each sentencing decisions. The effective connectivity of laypersons and legal experts is different in intensity and directionality, respectively, in decisions with and without mitigation (Fig. 5). When sentencing was based on reasoning about mitigating factors for a remorseful defendant, all bilateral connections between activated regions were significantly strengthened among laypersons (Fig. 5*A*, laypersons), but the intensity of these connections was low among legal experts; in particular, connectivity with the prefrontal region (BA32) was absent (Fig. 5*A*, legal experts). This difference in intensity implies that legal experts may have referred to the established reasons for mitigation and thus easily decided to reduce the punishment, whereas laypersons, who were not necessarily familiar with the standards for mitigation, showed increased strength in all connections, including the ones between the prefrontal and insula cortices.

Laypersons and legal experts also differed significantly when sentencing without mitigation, but in a different way. Laypersons showed intense connectivity between the prefrontal and insular cortices during sentencing both with and without mitigation. In this condition, legal experts also exhibited strengthened connectivity between the right anterior insula and the PFC, that is, BA10 rather than BA32, but in different directions from the ones of laypersons’. Between them, the effective connectivity was exactly opposite: from the right insula to other regions (including BA10) among laypersons (Fig. 4*B*, laypersons) and from BA10 to the emotion-related regions (including the right insula) among legal experts (Fig. 5*B*, legal experts).

Distinct connectivity from the emotion-related regions (i.e., BA23 and the insula) to the PFC (i.e., BA32 and BA10) might be implicated in regulation of emotion (Ghaziri et al. 2017). This implication is also supported by the individual-level correlation between connectivity strength and sentencing decisions (Fig. 6). Connectivity to BA10 and to BA32 was associated with fewer and more changes in punishment triggered by remorse, respectively. Stronger connectivity to BA10 had a restraining effect (Fig. 6*B*), but the connectivity to BA32 modulated the effect of emotion on decisions to change punishments based on the defendant’s remorse as a mitigating condition (Fig. 6*A*). This result also confirmed the distinction of roles between BA10 and BA32. BA10, along with BA32, regulates and integrates social and emotional stimuli (Amting et al. 2010). However, BA10 is more involved in emotionally charged dilemmas (Dampney 2018) in close connection with a wide range of regions, such as the precuneus, BA23, and insular cortex (Peng et al. 2018).

Taken together, the directionality of connections between the prefrontal region (BA10) and the anterior insular is reversed between laypersons and legal experts, and this reverse directionality is specific to decisions without mitigation. Legal scholars have also considered that decisions with and without mitigation are different in nature. In law practice, reducing punishment for a remorseful defendant is based on mitigation, and this occurrence is defined as a “mitigation” or “extenuation” problem. Without such reasoning, increasing punishment is considered more carefully. The results on effective connectivity are also consistent with observations that judges are more cautious to increase the punishment of a remorseless defendant than to decrease that of a remorseless one (Kawai 2011). Neural results, that is, strengthened connectivity from the PFC, indicate that legal experts consider reducing and increasing punishments for a remorseful or remorseless defendant, that is, with and without mitigation, respectively, to be fundamentally different decisions (Eisenberg et al. 1998).

The present study clarified the relevance of connectivity analysis (Elliott et al. 2020) by examining the neural circuitry associated with sentencing decisions, which overlaps with the circuitry of social and moral cognition (Funk and Gazzaniga 2009; Li et al. 2014). The different results in laypersons and legal experts indicated that lower and higher levels of expertise were plausibly associated with distinct patterns of effective connectivity. The neural results suggested that legal experts’ specialized training might have caused them to develop distinct effective connectivity from the PFC to the emotion-related regions. This result is expected to bring policy implications for the design of criminal justice systems to include the participation of laypersons in sentencing decisions.

## Author Contributions

RH and GN considered the concept of experiment; TA, HS, SO, JK designed the experiment; TA carried out the experiment; TA, HS, SO, and JK contributed data; TA and JK performed the analyses; SO and JK supervised the experiment; TA, HS, and JK wrote the paper; all authors evaluated and approved the manuscript.

## Notes

We would like to thank Dr. Kenichi Ueno (RIKEN CBS) for providing RETROICOR programs. We gratefully appreciate the excellent research assistance of Sumiko Ishida and Hiroko Nishikawa. We appreciate excellent comments from two reviewers. *Conflict of Interest*: The authors declare no competing interests.

## Funding

The University of Tokyo Edge Capital (UTEC, Grant Number: 1601-107); Japan Society for the Promotion of Science (Grant Number: 18H03612); Japan Society for the Promotion of Science (Grant Number: JP16H06280).

## Supplementary Material

SupplementaryFigureA_bhab484

SupplementaryFigureB_bhab484

SupplementaryTable_bhab484

SupplementaryText_bhab484

SupplementaryInformation_bhab484

SupplementaryDataParticipantAggregation_bhab484
