# Supplementary material for: Effective connectivity and criminal sentencing decisions: dynamic causal models in laypersons and legal experts
Source: Cereb Cortex. 2022 Jan 18;32(19):4304–16. doi: 10.1093/cercor/bhab484 (PMC9528897; doi:10.1093/cercor/bhab484)
Supplement: SupplementaryTable_bhab484 [file supplementarytable_bhab484.docx]

Supplementary **Table. Activation in other regions of the contrast of Punishment > Daily-Life**

**(A) When considering a sentence for a remorseful defendant (punishment reduction)**

| **Region** | **Side** | **BA** | **MNI coordinates** | | |  | **peak-level** | **cluster_level** |  |
| --- | --- | --- | --- | --- | --- | --- | --- | --- | --- |
|  |  |  | **x** | **y** | **z** | **T** | **P_FWE_corr** | **P_FWE_corr** | **k=** |
| **IFS** | **R** | **R BA40** | **50** | **-28** | **24** | **5.415** | **0.016** | **0.000** | **867** |
| **IFS** | **R** | **R BA40** | **54** | **-2** | **6** | **4.922** | **0.076** | **0.000** | **867** |
| **IFS** | **L** | **L BA40** | **-56** | **-32** | **16** | **4.533** | **0.131** | **0.003** | **369** |
| **IFS** | **L** | **L BA40** | **-56** | **-22** | **16** | **4.533** | **0.131** | **0.004** | **369** |

**(B) When considering a sentence for a remorseless defendant (punishment increase)**

| **Region** | **RL** | **BA** | **MNI coordinates** | | |  | **peak-level** | **cluster_level** |  |
| --- | --- | --- | --- | --- | --- | --- | --- | --- | --- |
|  |  |  | **x** | **y** | **z** | **T** | **P_FWE_corr** | **P_FWE_corr** | **k=** |
| **BA23** | **L** | **L BA23** | **-4** | **-44** | **26** | **6.012** | **0.003** | **0.000** | **2529** |
| **Iinsula1** | **R** | **R BA40** | **52** | **-26** | **20** | **7.325** | **0.000** | **0.000** | **2397** |
| **Insula2** | **R** | **R BA6** | **50** | **-6** | **10** | **6.876** | **0.000** | **0.000** | **2397** |
| **Insula3** | **R** | **R BA6** | **56** | **-2** | **6** | **5.895** | **0.004** | **0.000** | **2397** |
| **Insula1** | **L** | **L BA6** | **-50** | **0** | **-2** | **6.188** | **0.001** | **0.000** | **1714** |
| **Insula2** | **L** | **L BA6** | **-58** | **-4** | **12** | **5.743** | **0.007** | **0.000** | **1714** |
| **Insula3** | **L** | **L BA6** | **-56** | **-24** | **14** | **5.732** | **0.007** | **0.000** | **1714** |
