# Supplementary material for: Effective connectivity and criminal sentencing decisions: dynamic causal models in laypersons and legal experts
Source: Cereb Cortex. 2022 Jan 18;32(19):4304–16. doi: 10.1093/cercor/bhab484 (PMC9528897; doi:10.1093/cercor/bhab484)
Supplement: SupplementaryText_bhab484 [file supplementarytext_bhab484.docx]

**Supplementary Text: Script for five hypothetical cases**

**Case 1 (robbery-murder on the street).**

For the purpose of getting some pocket money, X and Y conspired to rob with knives a passerby of his/her wallet in the night. X and Y found V, an office worker, walking alone on the street. X and Y, respectively, pointed a knife at V, demanded money, and attempted to grab his wallet. However, V resisted fiercely and screamed for help. Therefore, both of X and Y instantly decided to kill V and stabbed him with knives. V got injured. X and Y ran away separately. As a result of the stab wounds, V died shortly after. The facts depicted above were proven by eyewitness accounts and surveillance camera recordings. [Participants were given 30 seconds to read.]

Q. Suppose X and Y were involved to the same extent in the murder of V. What do you think is the appropriate criminal punishment for X and Y? Please answer using a scale of 0 to 100.
[Participants were given 25 seconds to consider their response.]

Please move the arrow. [Participants were given 25 seconds to press the button to move the arrow on the scale to point to their response.]

1. X began to fear the severity of his/her conduct. X called an ambulance immediately after fleeing the scene and turned him/herself into the police. X honestly confessed everything about the crime and handed over to the police the knife with which he/she had stabbed V. X has offered the maximum possible compensation to V’s surviving family and has made a sincere apology at the trial for the crime he/she committed. [Participants were given 30 seconds to read.]

Q. What do you think is the appropriate criminal punishment for X? Please answer using a scale of 0 to 100. Your answer can differ from the one to the previous question. [Participants were given 25 seconds to consider their response.]

Please move the arrow. [Participants were given 25 seconds to press the button to move the arrow on the scale to point to their response.]

1. Y immediately threw the knife into the river to hide the evidence of his/her conduct. Y called X saying, “Never tell anything to anyone.” After being arrested, Y has made excuses without making any apology, such as “I was ordered and coerced by X,” “I didn’t use a knife,” and “I had no intention to kill him.” Y has even claimed, “It was self-defense. It was V who initiated a fight with us. I just resisted to protect myself.” [Participants were given 30 seconds to read.]

Q. What do you think is the appropriate criminal punishment for Y? Please answer using a scale of 0 to 100. Your answer can differ from the one to the previous question. [Scale omitted. Participants were given 25 seconds to consider their response.]

Please move the arrow. [Participants were given 25 seconds to press the button to move the arrow on the scale to point to their response.]

[Participants repeated the above process for Cases 2, 3, 4, and 5 below.]

**Case 2 (trespassing robbery-murder)**

X and Y, who were averse to working, conspired to steal money and goods. They broke into a house. When they were searching for valuables, the housewife V returned home and saw X and Y. V tried to call the police. X and Y, respectively, took out a knife, but V attempted to escape and continued to try to call the police. Therefore, both of X and Y decided to kill V, who had seen their faces. Both of X and Y stabbed her deeply in the neck and chest with knives. V got injured and died from loss of blood after being transported to a hospital. The facts depicted above were proven by a neighbor who saw the scene through the window. X and Y ran away separately when the neighbor ran to the scene after hearing the screams.

1. X immediately and deeply regretted his/her conduct and made a phone call to a friend. X said, “I did a terrible thing,” and asked the friend to call an ambulance. Accompanied by the friend, X turned him/herself into the police. X honestly confessed everything about the crime and handed over to the police the knife with which X had stabbed V. X has made a sincere apology at the trial and to V’s surviving family for the crime he/she committed.
2. Y immediately threw the knife into the river to hide the evidence of his/her conduct. After texting to X, “We should hide out separately for a while,” Y stayed at his/her friend’s place with a completely innocent face. Even after being arrested by the police, Y has made no apology. Y has made excuses, such as “I did not intend to kill her,” and “This result would never have happened if she hadn’t resisted.”

**Case 3 (fraud and murder)**

X and Y had been engaging in fraud to cheat victims out of money by making them sign a fake contract for high-interest financial products. One day, they attempted to cheat V at his/her house, but V said, “This is fraud. I recorded everything on this smart phone as evidence.” X and Y panicked and tried to persuade V, but V refused. X and Y thought they would be caught by the police if this continued. Therefore, both of X and Y decided to kill V. X and Y beat V alternately with an antique vase, which was at the scene. V died of a brain contusion. The facts depicted above were proven by the recording data on a small camera, which had been set by V in advance for the purpose of recording communications with X and Y.

1. X immediately and deeply regretted his/her conduct. X ran away at first, but X returned to V’s house in order to check on V’s condition, where he/she was found and arrested by the police. X honestly confessed everything about the crime and handed over to the police the knife with which X had stabbed V. X has made a sincere apology at the trial for the crime he/she committed and to V’s surviving family. X has returned all of the money to the victims of their past fraud and used all of X’s remaining wealth to compensate V’s surviving family.
2. While escaping to hide the evidence of his/her conduct, Y threw the vase into a garbage collection place. The vase has not yet been found. At the time of the arrest, which was based on X’s confession, Y claimed, “I don’t know X,” and “There must be some mistake.” Y has claimed, “There is no evidence. You haven’t found the vase,” even though V’s blood was found on Y’s clothing and hands. Y has made no apology.

**Case 4 (kidnap-murder)**

X and Y conspired to kidnap a child to demand a vast amount of ransom. To lure V into their car, they lied to V, who is a junior high school student with wealthy parents. They claimed, “Your parents had a traffic accident.” X and Y attempted to take V to a mountain lodge. However, V became suspicious about going onto a deserted mountain road and jumped out of the car when it slowed down. X and Y stopped the car to chase V. When V was cornered on a cliff, V tried to call the police with a cellphone. Therefore, both of X and Y instantly decided to kill V. Together they pushed V off the cliff, and V died due to the fall. The facts depicted above were proven by hikers who were watching the scene.

1. X immediately and deeply regretted his/her conduct. Immediately after driving back to the town and separating from Y, X turned him/herself into the police and honestly confessed everything about the crime. X has been fully cooperative in the interrogations, saying, “I took a valuable young life for nothing.” X has made a sincere apology for the crime he/she committed at the trial and to V’s parents. X has offered all of X’s savings as compensation.
2. Before they parted, Y threatened X, who began to regret the deed and was pale, by saying “If you tell the police, I will make you as a solitary murderer.” When the police visited Y’s home, based on X’s confession, Y claimed, “I don’t know anything at all.” Even after the police found eyewitnesses, Y claimed, without making any apology, “I didn’t do anything. X on his/her own decided to lure V and solely pushed V off the cliff.”

**Case 5 (domestic murder)**

X and Y reacted sharply to their parents’ strict parenting and performed poorly in high school. Eventually, they failed to enter college after finishing high school. One day, their parents said to them, “You two should leave this house tomorrow.” X and Y felt displaced. They got drunk and decided to kill their parents. X and Y, respectively, held a metal bat and beat their parents to death, while the parents were sleeping. The facts depicted above were proven by physical evidence, such as bloodstains on the bat, clothing, and the floor.

1. X immediately and deeply regretted his/her conduct. X left home with Y at first but returned soon. X was stunned and spent a few hours next to the bodies of their parents. Then, X voluntarily called the police to be arrested. X handed over the metal bat to the police. X has shown remorse for his/her selfishness and for not understanding his/her parents. X has sincerely regretted killing his/her parents for selfish reasons.
2. Y suggested to X, “Let’s go out separately and come back home tomorrow morning. Then, we can pretend that a robbery-murder has happened.” To hide the evidence of his/her conduct, Y left home immediately and hid the metal bat and blood-stained clothing in a clay pipe beside a river. Y was arrested when he/she returned home the next morning. Y has claimed, “It was X who initiated,” at the interrogations and has shown no remorse.

[Participants answered one daily (non-legal) question between each case above. Case A below is an example of the daily (non-legal) questions.]

**Case A (daily matter: temperature of coffee)**

X and Y were sitting and chatting in the lounge. They wanted to drink coffee, and so they went to the kitchen. When they were boiling the water for coffee, a colleague, V, came by. After a while, the water in the kettle boiled. X, Y, and V made and had coffee together. [Participants were given 20 seconds to read the case.]

What do you think is the temperature of this fresh coffee? Please answer using a scale of 0 degree to 100 degree Celsius. [Scale omitted. Participants were given 15 seconds to consider their answer.]

Please move the arrow. [Participants were given 20 seconds to press the button to move the arrow on the scale to point to their answer.]
