# Supplementary material for: Effective connectivity and criminal sentencing decisions: dynamic causal models in laypersons and legal experts
Source: Cereb Cortex. 2022 Jan 18;32(19):4304–16. doi: 10.1093/cercor/bhab484 (PMC9528897; doi:10.1093/cercor/bhab484)
Supplement: SupplementaryInformation_bhab484 [file supplementaryinformation_bhab484.docx]

**Supplementary Information: Remorse and Criminal Law**

In Japan where our experiment was conducted, for example, the language of the penal code does not explicitly refer to the defendant’s remorse nor the lack thereof, although judges often mention them in the reasoning part of judgment. The extenuating circumstance, which may include defendant’s remorse, is considered when judges decide whether to suspend execution of sentence (cf., Article 25 of Japan's Penal Code [JPC]) and whether to give a defendant commutation or exemption from punishment (cf., Articles 36-38, 66, 113, and 201 of JPC). The remorse of a *prisoner* is taken into consideration when deciding whether to grant parole or to shorten his/her prison term (cf., Article 28 of JPC).
