# Supplementary material for: Effective connectivity and criminal sentencing decisions: dynamic causal models in laypersons and legal experts
Source: Cereb Cortex. 2022 Jan 18;32(19):4304–16. doi: 10.1093/cercor/bhab484 (PMC9528897; doi:10.1093/cercor/bhab484)
Supplement: SupplementaryDataParticipantAggregation_bhab484 [file supplementarydataparticipantaggregation_bhab484.docx]

**Supplementary Data: Participant statistics**

1. Participants of GLM

| Group | male | female | Sum | average of age | standard deviation of age |
| --- | --- | --- | --- | --- | --- |
| Law students | 22 | 8 | 30 | 25 | 1.231764 |
| Law practitioner | 5 | 3 | 8 | 40.875 | 9.249517 |
| Non-law students | 19 | 9 | 28 | 21.32143 | 2.357864 |
|  | 46 | 20 | 66 |  |  |

1. Participants of DCM with mitigation condition

| Group | male | female | Sum | average of age | standard deviation of age |
| --- | --- | --- | --- | --- | --- |
| Law students | 19 | 8 | 27 | 24.92593 | 1.238048 |
| Law practitioner | 4 | 3 | 7 | 41.85714 | 9.529403 |
| Non-law students | 18 | 8 | 26 | 21.42308 | 2.402883 |
|  | 41 | 19 | 60 |  |  |

1. Participants of DCM without mitigation condition

| Group | male | female | Sum | average of age | standard deviation of age |
| --- | --- | --- | --- | --- | --- |
| Law students | 18 | 8 | 26 | 25.07692 | 1.163549 |
| Law practitioner | 5 | 3 | 8 | 40.875 | 9.249517 |
| Non-law students | 17 | 8 | 25 | 21.24 | 2.36784 |
|  | 40 | 19 | 59 |  |  |
